# S-Methylcysteine (SMC) Ameliorates Intestinal, Hepatic, and Splenic Damage Induced by *Cryptosporidium parvum* Infection Via Targeting Inflammatory Modulators and Oxidative Stress in Swiss Albino Mice

**DOI:** 10.3390/biomedicines8100423

**Published:** 2020-10-15

**Authors:** Ehab Kotb Elmahallawy, Gehad E. Elshopakey, Amira A. Saleh, Ahmad Agil, Ahmed El-Morsey, Dina M. M. EL-shewehy, Ahmed S. Sad, Tokuma Yanai, Walied Abdo

**Affiliations:** 1 Department of Zoonoses, Faculty of Veterinary Medicine, Sohag University, Sohag 82524, Egypt; eehaa@unileon.es; 2 Department of Biomedical Sciences, University of León (ULE), 24071 León, Spain; 3 Department of Clinical Pathology, Faculty of Veterinary Medicine, Mansoura University, Mansoura 35516, Egypt; gehadelshobaky@yahoo.com; 4 Department of Medical Parasitology, Faculty of Medicine, Zagazig University, Zagazig 44519, Egypt; amera.islam2011@yahoo.com; 5 Department of Pharmacology, Faculty of Medicine, University of Granada, 18016 Granada, Spain; aagil@ugr.es; 6 Parasitology and Animal Diseases Department, Veterinary Research Division, National Research Centre, 33 El Buhouth St. (former El-Tahrir St.), Dokki, P.O., Giza 18010, Egypt; el.el-sayed@nrc.sci.eg; 7 Zoology Department, Faculty of Science, Mansoura University, Mansoura 35516, Egypt; dinamagdy@mans.edu.eg; 8 Department of Pharmacology and Toxicology, Faculty of Pharmacy, Port Said University, Port Fuad 42526, Egypt; mosa1200@yahoo.com; 9 Laboratory of Wildlife and Forensic Pathology, Biomedical Science Examination and Research Center, Department of Veterinary Medicine, Faculty of Veterinary Medicine, Okayama University of Science, 1-3, Ikoinooka, Imabari 794-0085, Ehime, Japan; 10 Department of Pathology, Faculty of Veterinary Medicine, Kafrelsheikh University, Kafr el-Sheikh 33516, Egypt; waliedsobhy@yahoo.com

**Keywords:** *Cryptosporidium parvum*, hepatic damage, inflammatory biomarkers, intestinal injury, s-Methylcysteine

## Abstract

Cryptosporidiosis has been proposed to be one of the major causes of diarrhoeal disease in humans worldwide that possesses zoonotic concern. Thereby, this study investigated the potential effects of s-Methylcysteine (SMC) on the parasite in vivo followed by the measurement of cytokines, oxidative stress parameters, and an investigation of the major histopathological changes. Sixty male Swiss albino mice weighing 20–25 g were allocated equally into five groups and orally administered saline only (control), SMC only (SMC50) (50 mg/kg b.w.), and 10^4^ *Cryptosporidium parvum* oocysts per mouse via an esophageal tube (C + ve untreated). The fourth and fifth groups (C + SMC25, C + SMC50) administrated 10^4^ *C. parvum* oocysts combined with SMC25 (low dose) and 50 (high dose) mg/kg b.w., respectively. At days 7 and 14 post-infection (PI), the feces was collected from each group in order to count *C. parvum* oocysts. After two weeks of treatment, the animals were euthanized and the serum was collected for biochemical analysis. Next, the intestinal, spleen, and liver sections were dissected for histopathological examination. The results revealed lower oocyst numbers in the C + SMC25 and C + SMC50 groups compared to the infected untreated group. Moreover, higher doses of SMC treatment significantly reduced the enteritis induced by *C. parvum* in a dose-dependent manner. The hepatic lesions were also mitigated as demonstrated in C + SMC25 and C + SMC50 groups unlike the infected group via lowering the serum alanine aminotransferase (ALT), aspartate aminotransferase (AST), and alkaline phosphatase (ALP) enzymes and increasing albumin and globulin serum levels. SMC administration also reduced cytokines production (SAP, TNF-α, IL-6, and IFN-γ) mediated by *Cryptosporidium* infection in contrast to the infected untreated group. There were marked lymphoid depletion and amyloidosis observed in the infected untreated group, while the treated groups showed obvious increase in the lymphoid elements. Moreover, the scoring of intestinal parasites, hepatic, and splenic lesions in the SMC-treated groups exhibited significantly lower pathological lesions in different organs in a dose-dependent manner, compared to the infected untreated group. Our results also revealed a significant change in the malondialdehyde content with an elevation of glutathione and superoxide dismutase in the intestines collected from C + SMC25 and C + SMC50 mice relative to the untreated group. Taken together, our results indicated that SMC could be a promising effective compound for treating and declining *C. parvum* infestation via restoring structural alterations in different tissues, enhancing antioxidant enzymes, and suppressing the cytokines liberation.

## 1. Introduction

Enteric infections remain a major significant public health concern in developing and developed nations [1,2,3]. Among others, numerous outbreaks of zoonotic cryptosporidiosis have been described as major protozoan waterborne cause of diarrhoea in humans worldwide [1,4,5]. This waterborne disease is caused by leading unicellular protozoan of the genus *Cryptosporidium* [5,6]. Among all identified species of this protozoan, *Cryptosporidium parvum* has been considered an important zoonotic species with a wide epidemiological profile that includes multiple hosts and reservoirs [4,6,7]. Humans might contract the infection mainly through the fecal–oral route via ingestion and less likely through inhalation of coughed on fomites [5,8]. The parasite comprises of three developmental stages that include meronts, gamonts, and oocysts. Taken into account, the later one can survive for a long period outside the host and resist disinfectants as a result of the strong protective effect of its oocyst wall [6]. The clinical impact of the disease ranges from mild to severe or long-term non-bloody diarrhea but the infection could be recurrent in some immunocompetent hosts and become life-threatening in immunocompromised individuals such as acquired immunodeficiency syndrome (AIDS) patients and those who receive immunosuppressive drugs [9,10]. Additionally, cryptosporidiosis might result in significant morbidity and mortality in human, particularly among children living in resource-poor settings in developing countries [11,12,13,14,15].

The diagnosis of the disease is usually accomplished through examination of stool for the presence of oocysts, morphometric identification which is often difficult, and via a combination of methods incorporating data from morphometric, molecular techniques, and host specificity; however, this later method is usually encountered with many limitations as a result of cost, duration, expertise, or reliability [16]. One of the main control strategies of the disease is the development of novel drug targets for combating the diseases [17]. Despite innumerable studies on developing novel drug targets against the cryptosporidiosis, no effective chemotherapy is available and the treatment mainly relies on using combined therapy using paramomycin with protease inhibitors or recombinant Interleukin 12 (IL-12) or Nitazoxanide [6,17,18,19,20]. However, this drug seems to be ineffective in human immunocompromised individuals and the use of paramomycin has been proved to be suppressive in specific cases [18,19]. Clearly, a complete removal of the parasite from the host remains the main challenge in order to control the disease in the host.

It is noteworthy to mention that s-Methylcysteine (SMC) is one of the main organosulfur compounds that widely occur in many edible vegetables such as garlic [21,22,23]. Interestingly, several previous reports have documented a wide range of activities of garlic and its components, particularly SMC, for treatment of various physiological and pathological conditions [24,25,26]. In this regard, some previous reports revealed that garlic, SMC, and other organosulfur compounds of raw or extracted garlic showed anticancer [27], antioxidant [28], anti-inflammatory [29], antidiabetic [30], cardioprotective [24], neuroprotective [31], hypocholesterolemic [30], antiinfective [32,33], and hepatoprotective effects [34,35,36]. To the authors’ knowledge, no former investigations assessed the effect of SMC against *C. parvum* either in vitro or in vivo. Hence, the present study was undertaken to assess the effect of SMC against the parasite in vivo in an endeavour to develop a novel drug against the parasite. The study also involved the measurement of various oxidative stress parameters and an investigation of the major histopathological changes following administration of the drug.

## 2. Materials and Methods

### 2.1. Ethical Considerations

The ethical approval of the present study was obtained from a guidance of the Research, Publication, and Ethics Committee of the Faculty of Veterinary Medicine, Kafrelsheikh University, Egypt, which complies with all relevant Egyptian legislations in publication and research. The ethical approval number is KFS-2019/1. 

### 2.2. Materials

S-Methylcysteine, as well as other chemicals and reagents were purchased from Sigma-Aldrich (Cairo, Egypt). All materials were of analytical grade and used as received.

### 2.3. Parasite Material and Parasite Preparation 

*Cryptosporidium parvum* oocysts were purchased from the Parasitology Department at the Theodor Bilharz Research Institute and were stored at 4 °C in potassium dichromate 2.5% solution (*w/v*) in the Department of Pathology, Faculty of Veterinary Medicine, Kafrelsheikh University, following the protocol described elsewhere [37]. The oocysts of the parasite were washed shortly before use three times in distilled water for removal of potassium dichromate, then centrifuged at 1500×·*g* for 10 min, and the organisms were counted with a hemocytometer. Later on, the suspension contained approximately 10^4^ oocysts/mL which is required for the infection of mice and was prepared by dilution of the organism in the appropriate amount of distilled water [38].

### 2.4. Animals and Experimental Protocol

As mentioned above, the present work was conducted in compliance with the Egyptian guidelines for animal care, handle, and protection. Male Swiss albino mice (fa/fa; 20–25 g body weight (b.w.); *n* = 60) at the age of 6 weeks were purchased from Animal house (National Research Centre, Giza, Egypt) and housed in clean well-ventilated cages under standard laboratory conditions (synchronized to a 12-h light/dark cycle and at a temperature-controlled room of 24–28 °C). Animals were fed on a standard diet (pelleted food and water) and provided water ad libitum. Furthermore, they were used for the experiments after one week of the acclimatization period.

**Figure 1:**
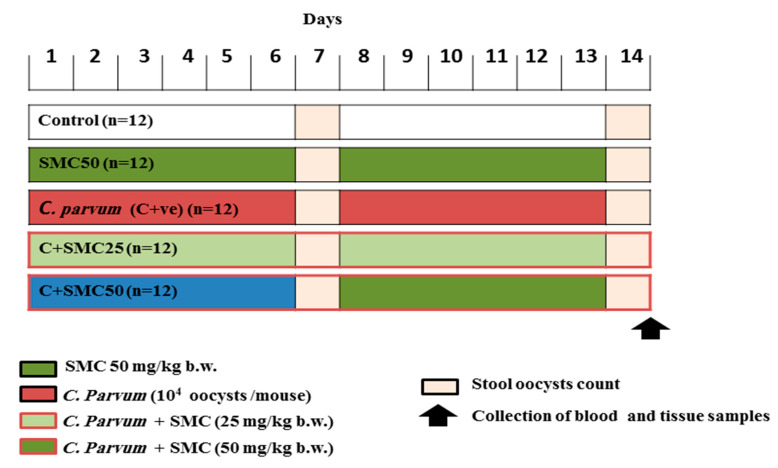
Treatment protocol and doses of used s-Methylcysteine (SMC).

At the age of 7 weeks, the animals were divided into 5 groups of 12 mice each as follows: the first group was administered saline (negative control group), the second group (SMC50) was only administered s-Methylcysteine at a dose of 50 mg/kg b.w., the third group (C + ve, infected untreated) was control positive and experimentally infected by 10^4^ *C. parvum* oocysts per mouse using an esophageal tube. Importantly, the dose of SMC was determined according to a protocol that is described elsewhere [24]. The infected group and C + SMC groups were given dexamethasone sodium phosphate at a dose of 125 μg/day for 14 consecutive days prior to oocysts inoculation and throughout the experimental protocol for induction of immunosuppression in infected animals following the protocol described elsewhere [39]. In accordance with the 4th and 5th group (C + SMC25, C + SMC50), they were given 10^4^ *C. parvum* oocysts by intragastric inoculation combined with s-Methylcysteine-treated groups at a dose of 25 (low dose) and 50 (high dose) mg/kg b.w., respectively. The treatment protocol is summarized in Figure 1. 

Examination of stools of mice was done microscopically using direct wet saline smear and iodine stained with acid-fast (AF) stain to detect the presence of *Cryptosporidium* oocysts. After two weeks of treatment, the animals were euthanized with diethyl ether inhalation and the collection of blood samples was done without anticoagulants. The obtained serum was stored at −20 °C for further biochemical analysis. Intestinal tissue sections were collected from different animal groups treated to be used in antioxidant/oxidative stress markers evaluation. Other intestine, spleen, and liver sections were dissected and sent to the Department of Pathology, Faculty of Veterinary Medicine, Kafrelsheikh University, Egypt, for further histopathological examination. 

### 2.5. Measurement of Serum Biochemical Parameters

Measurement of aspartate aminotransferase (AST), alanine aminotransferase (ALT), albumin, globulin, and alkaline phosphatase (ALP) were determined using commercially available diagnostic kits according to the manufacturer’s instructions (LifeSpan Biosciences, Seattle, WA, USA). Serum Amyloid P concentration was measured by sandwich ELISA using rabbit SAP serum (US Biological, Salem, MA, USA). Furthermore, the level of Tumor Necrosis factor alpha, Interferon gamma (IFN-γ), and Interleukin 6 (IL-6) were analyzed by the sandwich ELISA kit RayBiotech (Peachtree Corners, GA, USA). 

### 2.6. Histopathological Examination

Samples from intestine, spleen, and liver of different animal groups were trimmed, fixed in neutral buffered formalin (10%), dehydrated, cleared, and embedded in paraffin wax. The hard paraffin blocks were sliced into sections of 5 µm in thicknesses for each organ to make a ribbon of tissue that was then stained with hematoxylin and eosin (H&E) according to the protocol described elsewhere [40]. The histological examination of stained slides of the different groups was blindly examined using a light microscope for quantitative assessment of the histopathological changes. 

The reported histopathological lesions were scored upon a 5 points scale which scored from 0 to 5. The intestinal lesions were assessed on the basis of presence enteritis features as crypt degeneration, necrosis, desquamation, hyperplasia, and inflammation. Moreover, the number of oocysts within the intestinal mucosa was counted and expressed as the number/mm^2^. The hepatic lesions were assessed according to the degree of congestion, degeneration, apoptosis, necrosis, and granuloma formation. Lesions on spleen were scored according to congestion, lymphoid depletion, and amyloid deposition. 

### 2.7. Evaluation of Oxidative Stress and Antioxidant Markers

Following intestinal tissues homogenization in phosphate buffer saline and centrifugation at 5000 rpm for 30 min, the resulting supernatants were used to measure the concentration of the oxidative stress marker, malondialdehyde (MDA) and the activity of antioxidant enzyme, superoxide dismutase (SOD), and glutathione (GSH) as previously described (Biodiagnostics, Giza, Egypt) [41]. 

### 2.8. Statistical Analysis

The values were expressed as mean ± SD and the data were statistically analyzed by one-way ANOVA using the GraphPad PRISM software v.5 (La Jolla, CA, USA). The statistical significance variance was considered when *p* < 0.05.

## 3. Results 

### 3.1. Effects of s-Methylcysteine (SMC) on Shedding of Cryptosporidium Oocysts 

**Figure 2:**
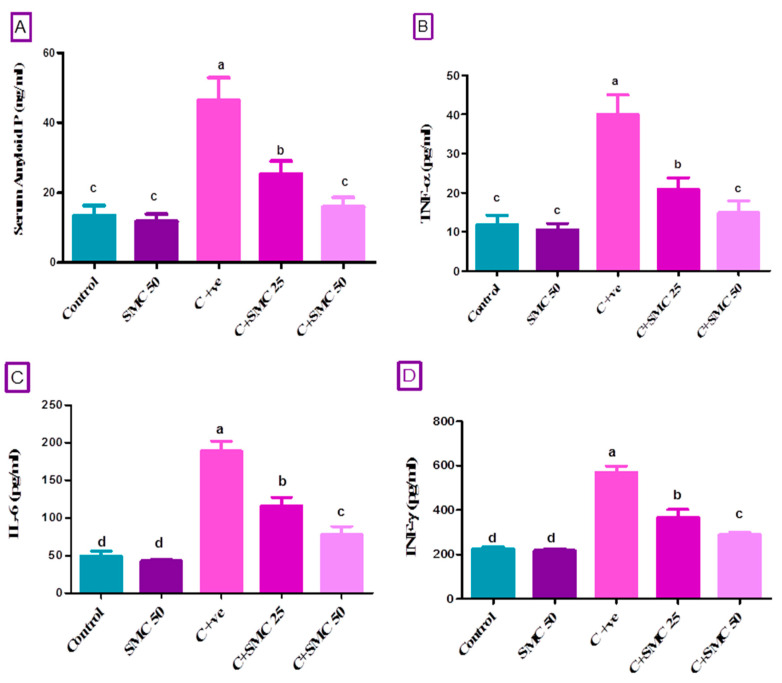
Serum level of amyloid P (**A**), Tumor Necrosis factor (TNF-α, (**B**)), Interleukin 6 (IL-6, (**C**)), and Interferon (IFN-γ, (**D**)) after two weeks of s-Methylcysteine (SMC) treatment in *C. parvum-*infected mice. Control (received saline, orally); SMC50 (treated with s-Methylcysteine, 50 mg/kg, orally); C+ ve (infected with 10^4^ *C. parvum* oocysts); C + SMC25 (infected and treated with s-Methylcysteine, 25 mg/kg, orally); C + SMC50 (infected and treated with s-Methylcysteine, 50 mg/kg, orally). Data were expressed as mean ± S.D. Each bar carrying different letters (a, b, c, d) is significantly different (*p* < 0.05).

Shedding of *C. parvum* oocysts (Table 1) was observed in control, infected non-treated (C + ve) and SMC-treated groups (SMC50, C + SMC25, and C + SMC50 groups) at days 7 and 14 post-infection (PI) (Figure 2). The mean oocyst number shed in stools of both in the C + SMC25 and C + SMC50 groups was significantly reduced (*p* < 0.05) at days 7 and 14 PI when compared to the infected non-treated group. The lowest oocyst number (*p* < 0.05) was recorded in the C + SMC50 group counterweight to the infected non-treated and C + SMC25 groups. Feces of control and SMC50 groups were still negative for *C. parvum* oocysts.

### 3.2. SMC Attenuates Cryptosporidium-Induced Liver Damage

The levels of serum hepatic enzymes (ALT, AST, and ALP), albumin, and globulins were measured as biomarkers for liver damage at two weeks PI with *C. parvum* along with the SMC treatment. As shown in Table 1, *Cryptosporidium* infection (infected group) exhibited marked liver damage indicated by an increased serum of ALT, AST, and ALP levels (*p* < 0.05) compared to the control group. Meanwhile, the concomitant SMC treatment, especially the higher dose, significantly reduced (*p* < 0.05) hepatic damage as demonstrated in the C + SMC25 and C + SMC50 groups unlike the infected untreated group.

Proteinogram assay illuminated the significant lowest level (*p* < 0.05) of albumin and globulin in the infected non-treated group relative to the control. C + SMC25- and C + SMC50-treated mice revealed a significant increase in serum albumin and globulin (*p* < 0.05) counterweight to the infected non-treated group. Moreover, the highest globulin level (*p* < 0.05) was demonstrated in the C + SMC50 group in contrast to all other groups including control (Table 2).

### 3.3. SMC Modulates Cryptosporidium-Induced Inflammation

To assess the mechanisms involved in the progression of cryptosporidiosis, the serum amyloid P (SAP) and pro-inflammatory cytokines (TNF-α, IL-6, IFN-γ) were estimated (Figure 2). The data analysis of amyloid P, TNF-α, IL-6, and IFN-γ indicated a significant elevation (*p* < 0.05) of their level in *Cryptosporidium*-infected mice relative to the control one. The concentrations of SAP and all pro-inflammatory cytokines were significantly lower (*p* < 0.05) in infected SMC-treated mice (C + SMC50 and C + SMC25 groups, respectively) than that of untreated infected mice. The treatment of *Cryptosporidium-*infected mice with a higher dose of SMC (50 mg/kg) resulted in a significant reduction (*p* < 0.05) of SAP and TNF-α to their normal level when compared to the control group (Figure 2A,B).

### 3.4. Histopathology

**Figure 3:**
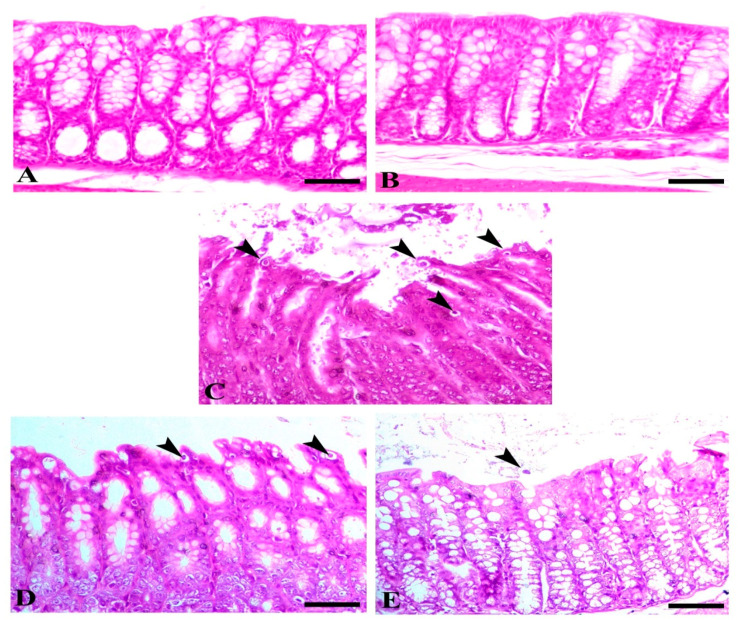
Intestine of different animal groups. (**A**) Control, (**B**) sham SMC50, (**C**) C + ve (arrowheads indicate numerous parasitic oocysts), (**D**) C + SMC25, and (**E**) C + SMC50 both intestinal sections showing a marked decrease of the intestinal oocyst (arrowheads). Hematoxylin and eosin (H&E) stain, bar = 50 µm.

Microscopically, the colonic mucosa of both control and sham groups showed normal intestinal crypts. Intestine of C + ve showed a feature of chatarahal entritis associated with a parasitic invasion of the epithelium of the glands, marked dequmative changes, and hyperplastic changes within the basal aspect of the crypts. The infected animals and animals treated with SMC at both doses revealed dose-dependent retrival of the normal intestinal mucosa as demonstrated with the decrease of parasitic cysts, enteritis, and hyperplastic lesions (Figure 3). 

**Figure 4:**
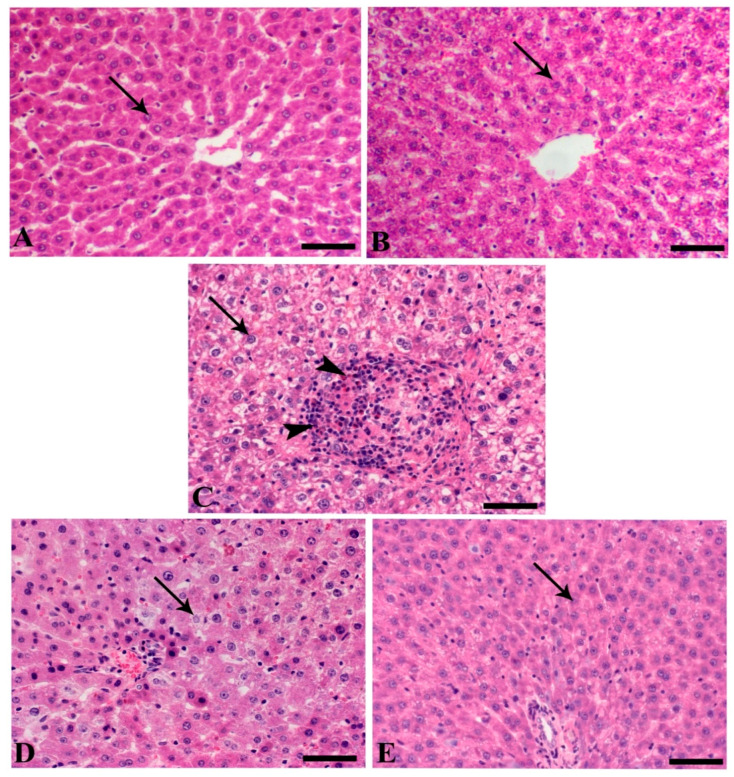
Liver of different animal groups. (**A**) Control, (**B**) sham SMC50 (arrows indicate normal hepatocytes), (**C**) C + ve (arrowheads indicate granuloma-like lesion rich in a high number of lymphocytes, macrophages, and eosinophils), (**D**) C + SMC25 (arrow reveals a decrease in hepatic vacuolation), and (**E**) C + SMC50 (arrow indicates mild hepatic vacuolation). H&E stain, bar = 50 µm.

**Figure 5:**
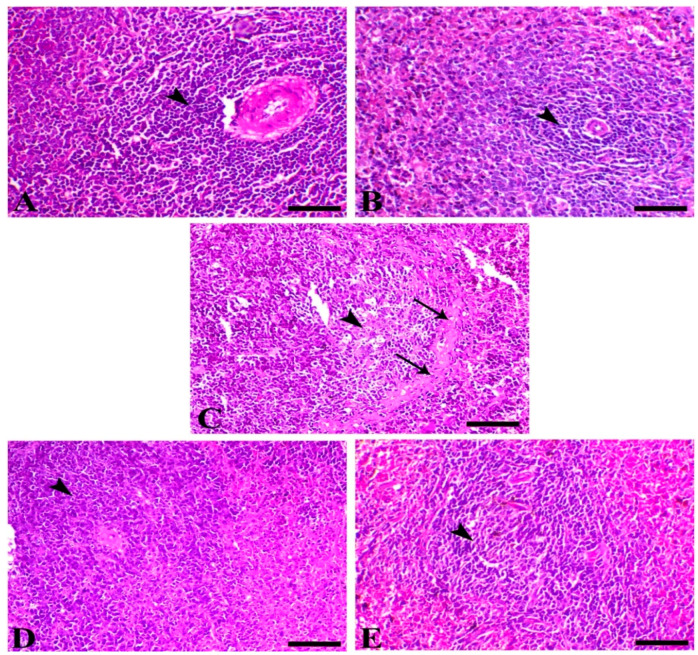
Spleen of different animal groups. (**A**) Control, (**B**) sham SMC50 (arrowheads indicate normal lymphoid follicle filled with numerous lymphocytes), (**C**) C + ve (arrowhead indicates marked lymphoid depletion and arrows indicate amyloid deposition), (**D**) C + SMC25, and (**E**) C + SMC50 both splenic sections showing marked improvement of lymphoid content (arrowheads). H&E stain, bar = 50 µm.

The liver of control and SMC50 showed normal hepatic tissues represented mostly with normal hepatic cells that arranged normally around the central vein. The liver of C + ve animals showed multiple necrotic foci within the hepatic tissues associated with marked aggregation of inflammatory cells as lymphocytes, macrophages, and esinophils give the picture of a granulomatous reaction. Furthermore, most of the hepatocytes revealed hydropic vacuolar changes. The hepatic tissues of infected animals treated with SMC25 demonstrated a marked decrease in the necrotic changes with few vacuolion and mild apoptosis of the hepatoctes (Figure 4). The spleen of both the control and sham group demonstrated normal lymphoid follicles formed of lymphoid cells aggregation around central arteriole, while the infected mice showed a severe degree of lymphoid depletion associated with a deposition of amyloid substances, mostly on outer the follicles (Figure 5).

**Figure 6:**
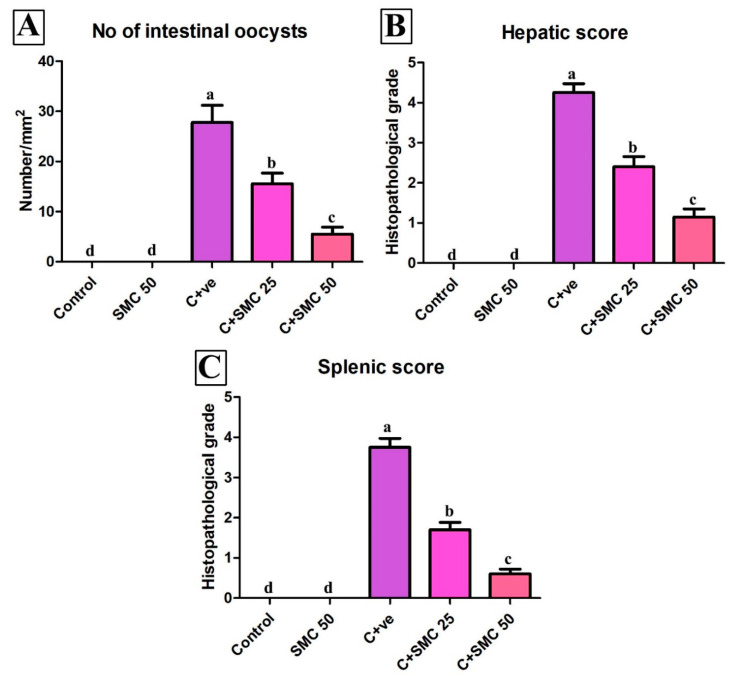
Quantitative scoring of intestinal oocyst count (**A**), hepatic (**B**), and splenic (**C**) lesions. Data were expressed as mean ± S.D. Each bar carrying different letters (a, b, c) is significantly different (*p* < 0.05).

Interestingly, the quantitative scoring of the intestinal parasites, hepatic, and splenic lesions showed a marked significant decrease of the pathological lesions within the different organs in a dose-dependent manner (*p* < 0.05) (Figure 6).

### 3.5. SMC Restores Intestinal Oxidative Injury Generated by Cryptosporidium

**Figure 7:**
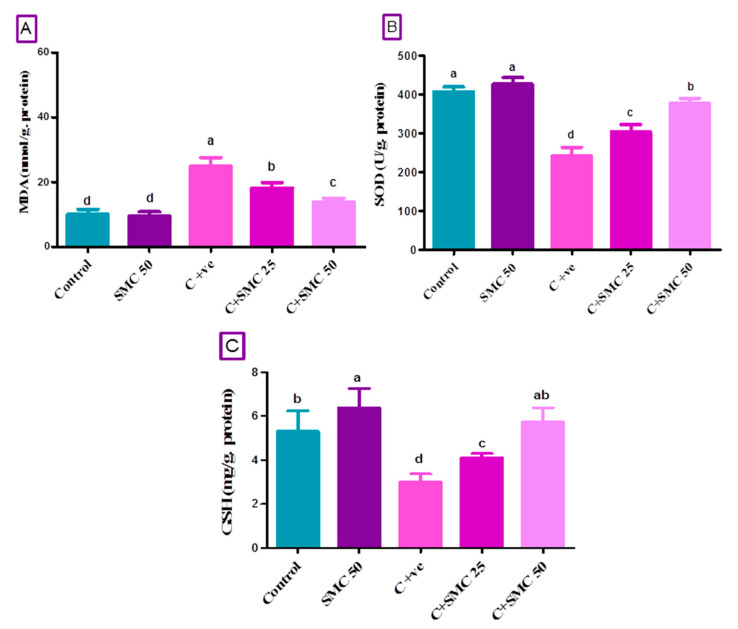
Intestinal malondialdehyde (MDA, (**A**)), superoxide dismutase (SOD, (**B**)), and glutathione (GSH, (**C**)) after two weeks of s-Methylcysteine (SMC) treatment in *C. parvum*-infected mice. Control (received saline, orally); SMC50 (treated with s-Methylcysteine, 50 mg/kg, orally); C + ve (infected with 10^4^ *C. parvum* oocysts); C + SMC25 (infected and treated with s-Methylcysteine, 25 mg/kg, orally); C + SMC50 (infected and treated with s-Methylcysteine, 50 mg/kg, orally). Data were expressed as mean ± S.D. Each bar carrying different letters (a, b, c, d) is significantly different (*p* < 0.05).

*Cryptosporidium* infestation impaired antioxidant complex activities, which led to a generation of ROS. *C. parvum* induced oxidative stress by an elevation (*p* < 0.05) of malondialdehyde (MDA) content and a reduction (*p* < 0.05) of SOD activities and GSH level in mice intestines as demonstrated in the infected untreated group compared to the control group (Figure 4). On the other hand, the treatment of mice with SMC only, C + SMC50, and C + SMC25 groups significantly corrected (*p* < 0.05) the levels of MDA, GSH, as well as SOD activity toward the control level in a dose-dependent manner, when compared to both infected and control groups (Figure 7).

## 4. Discussion

Despite the considerable improvement in public sanitation services, enteric infections caused by protozoan parasites remain a major risk to human and animal health as potential zoonotic pathogen. Among others, cryptosporidiosis is an enteric infection with worldwide distribution, particularly in countries with inadequate sewage treatment and poor water quality [11,42,43]. The lack of vaccines and effective drugs against the disease represents a significant limiting factor in disease control [42]. Clearly, developing new drugs and vaccines that target parasites are urgently needed. 

S-Methylcysteine (SMC) is a naturally active component of many *Allium* plants such as onion, garlic, and leek [21,22,23]. Previous studies investigated the anti-protozoal activity of garlic extracts and its phytochemicals against several protozoal diseases including cryptosporidiosis [44]. However, our study is considered the first to report the protective effects induced by s-Methylcysteine against the intestinal hepatic, splenic, and hepatic damage resulting from the infection by *C. parvum*.

Our findings showed significant dose-dependent alleviation of stool oocyst counts with the administration of SMC. High and low doses of SMC (C + SMC50 and C + SMC25) significantly minimized the *C. parvum* oocysts count in experimentally infected mice, particularly with the higher dose (SMC, 50 mg/kg). Similar to our results, garlic successfully extirpated the *Cryptosporidium* oocysts from the stool and intestinal sections of the infected immunocompetent mice treated with garlic for two weeks [33]. The garlic oil was also proved to have a broad-spectrum of anti-parasitic activity against certain microorganisms such as *Trypanosoma, Cochlospermum planchonii*, *Leishmania, Plasmodium*, and *Giardia* [32]. The antiprotozoal effect of garlic belongs to the presence of several phytochemicals including allicin and several organosulfur compounds as *N*-acetylcysteine that possess antimicrobial activity via enhancing phagocytosis and stimulating the natural killer cells activity [45,46]. The serum elevation of alanine aminotransferase (ALT) signalizes the cell membrane injury while aspartate aminotransferase (AST) refers to mitochondrial damage of hepatic tissue [47]. In addition, alkaline phosphatase (ALP) is used as an indicator of hepatobiliary disease and hepatic cellular damage [48]. In our study, an increased level of these enzymes was demonstrated which indicated hepatocyte damages mediated by the *C. parvum* infection in mice. Moreover, a marked reduction in albumin and total globulins levels was presented in *C. parvum*-infected mice. 

Our findings corroborate with the previous results of Aboelsoued et al. [49] who recorded an elevation in ALT and AST in experimentally infected mice with *Cryptosporidium.* These results emphasized the extra-intestinal harmful effect of *Cryptosporidium* infection [50]. Moreover, the production of reactive oxygen species (ROS) was clearly implicated in the pathogenesis of experimental *C. parvum* infection in mice and induced oxidative stress damage in hepatic tissue [51]. The reduction in the albumin level may be attributed to hepatocellular damage, as liver is the main organ responsible for its synthesis [52]. Additionally, it was previously reported that the level of γ globulin was markedly reduced while albumin was insignificantly affected in *Cryptosporidium* infection [53]. In this study, the serum level of liver enzymes (ALT, AST, and ALP), albumin, and globulin were restored toward the control level referring to the suppressive effects of SMC against *Cryptosporidium* infestation. The high dose of SMC (50 mg/kg) strongly ameliorated the harmful effects of *C. parvum* by minimizing hepatocyte damage and decreasing serum concentrations of the above enzymes. This amelioration may be correlated to the presence of organosulfur compounds as S-allylcystein (SAC) in garlic has various antioxidant properties via inhibiting lipid peroxidation and has the ability to diminish the histological damage in liver of mice mediated by carcinogenic drugs [54,55,56] 

Serum amyloid P (SAP, pentraxin-2) is a member of C-reactive protein that regulate numerous aspects of the innate immune system [57]. Pro-inflammatory cytokines, such as IFN-γ, IL-6, and TNF-α, are commonly secreted by activated macrophages and are included in the inflammation up-regulation process [58]. IFN-γ triggers the M1 macrophages expansion, ROS and NO elicitation, and apoptosis [59]. The critical roles of IFN-γ, TNF-α, and NO in the host resistance against parasite infestation have been clarified in other studies [60,61]. Our results exhibited an increase in pro-inflammatory cytokines secretion (TNF-α, IL-6, IFN-γ) in infected non-treated mice compared to control ones, indicating the inflammatory responses attributed to *Cryptosporidium* infestation. Earlier investigations found that α1-globulin is significantly elevated in rats infected with *C. parvum* [53] through an increasing level of serum amyloid A and/or serum amyloid P that were previously confirmed to be increased during infection [62] and inflammation [63]. Interestingly, a landmark study has revealed a significant increase in the expression of TNF-α, IL-6 genes in leukocytes of *C. parvum*-infected mice [64]. In accordance with previous data, the immune response during *Cryptosporidium* infection presented by an increasing expression of immune mediators such as TNF-α, IL-6, IFN-γ by T-helper cell (Th1) particularly protect against intracellular infections including *C. parvum* [65]. Furthermore, TNF-α together with IFN-γ helps to activate macrophages to release huge amounts of IL-12 to control parasite replication during early *Cryptosporidium* infection [66].

In the present work, the treatment of infected mice with SMC decreased the release of SAP, TNF-α, IL-6, and IFN-γ, especially with the higher dose (50 mg/kg) that could return SAP and TNF-α to their normal level. SMC employs its anti-inflammatory activities through suppressing the expression of NF-kB p65 and NF-kB p50, restricted p38 phosphorylation, and reduced inflammatory mediators. Moreover, SMC effectively inhibited cyclooxygenase-2 (COX-2)/prostaglandin E(2) (PGE(2) pathways which are considered potent mediators in several inflammatory diseases [67]. Another mechanism is that aged black garlic (ABG) extract could act by prohibiting the inducible nitric oxide synthase (iNOS) and COX-2 expression, and, consequently, prevented IL-6 and TNF-α formation [68]. Additionally, the oral administration of SMC could significantly decrease the plasma level of TNF-α in diabetic rats [29]. 

Regarding the histopathological findings, several previous studies documented various pathological lesions induced by the infection by *Cryptosporidium* spp. in immunocompromised and immunocompetent hosts [69,70,71,72]. In this concern, a high-grade of cell dysplasia in the liver and ileum with the evidence of dysplastic changes in the bile has been demonstrated previously by Certad et al. [73] and Abdou et al. [74]. The hepatotoxicity was previously confirmed in our results by elevation of serum liver enzymes. Microscopic examination of *Cryptosporidium-*infected ileum showed atrophy, degeneration, and necrosis with sloughing of villi upper tips. Thickening and flat villi with inflammatory cellular infiltration in submucosa and lamina propria were also detected [53]. The amelioration of the intestinal inflammation mediated by garlic aqueous extract has been suggested by Gaafar [33] who found a decrease in myeloperoxidase (MPO) activity, a reliable index of inflammation intensity, which is coincided with altered mucosal architecture, blunting, widening, and shortening of the intestinal villi in case of cryptosporidiosis. Restoring the histopathological alterations in liver, spleen, and intestine architectures in response to the SMC treatment were reliable even in terms of biochemical and inflammatory markers. The strategy of SMC to restore tissue architecture may be correlated to the ability of garlic organosulfur compounds as S-allylcystein (SAC) to reduce lipid peroxidation and oxidative stress damage through scavenging O_2_^-^, H_2_O_2_, and HO. They also have anti-inflammatory effects through inhibition of iNOS expression in macrophages to regulate NO production [75,76]. 

Incrimination of ROS production has been reported in many pathological diseases infecting the gastrointestinal tract like inflammatory bowel disease [77]. The known function of SOD is catalyzing the toxic superoxide radical into less toxic hydrogen peroxide [78]. The highly considerable thiol antioxidant is GSH that present in the blood and different tissues and showed a major role in the detoxification of varied toxic compounds like xenobiotics and carcinogens and subsequently preserved the protein structure of the cell [79]. In the current study, C*. parvum* generated oxidative stress by elevation of MDA content and reducing SOD activity and the GSH level in the infected mice. A significant decline in the values of glutathione peroxidase (GP_X_) and total antioxidant capacity was also reported in *C. parvum*-infected mice [49]. The oxidative injury following C*. parvum* infestation was previously reported by other researchers who exhibited a decrease in GSH level and SOD activity [51,80,81]. This data emphasizes the oxidative stress linked to *Cryptosporidium* infection, suggesting that dealing with infection creates an overload on the animal that could exaggerate the oxidative injury [82]. 

Treatment with SMC modified the *Cryptosporidium*-generated oxidative damage by improving antioxidant content (SOD and GSH) and reducing the MDA level in intestinal tissue. GSH- and GSH-dependent enzyme, GPx, could be restored after SMC treatment in diabetic rats explaining the antioxidant property of SMC and its ability to conserve the cell components from free radical injuries [29]. Furthermore, The potential antioxidant effect of garlic extract was clarified in a previous report, that demonstrated an increase of SOD activity and other antioxidants due to the presence of different flavonoids and sulfur compounds as SMC in garlic that have high radical scavenging activities [44]. This effect may be correlated to the ability of SMC to inhibit NADPH oxidase pathway through down-regulation of p^47phox^ and gp^91phox^ expressions, which are components of the NADPH oxidase enzyme, subsequently depressing ROS production [67]. Surprisingly, Abdel-Daim et al. [83] revealed that Diallyl sulfide, one of the garlic sulfur compounds, exerts its antioxidant property by stimulating the mRNA expression of Nrf2 and the heme-oxygenase 1 enzyme.

## 5. Conclusions

Our study investigated the impacts of cryptosporidiosis on the intestine, spleen, and liver of mice. Additionally, the study corroborated the role of SMC (at doses of 25 and 50 mg/kg b.w.) in attenuating *C. parvum-*induced hepatic, splenic, and intestinal damage consequences, via enhancing the ability of antioxidant enzymes and suppressing the release of inflammatory mediators. 

Indeed, our results emphasize the importance of SMC co-administration as an effective and safe therapy for treating and declining *C. parvum* infestation. However, more investigation should be applied before approving SMC either alone or in association with other available drugs for the treatment of cryptosporidiosis. Our study also suggests that further future research seems mandatory to explore more mechanistic actions and pathways underlying the effects of SMC in cryptosporidiosis and the role of remaining reactive oxygen and nitrogen species in various organs, which is important for combating this disease of zoonotic importance.

## Figures and Tables

**Table 1:** The number of *Cryptosporidium parvum* oocysts shed in mice feces collected from different experimental groups at days 7 and 14 post-infection (PI).

Days Post Infection	Experimental Groups				
	Control	SMC50	C + ve	C + SMC25	C + SMC50
Day 7	0.00 ± 0.00 ^d^	0.00 ± 0.00 ^d^	41.20 ± 3.11 ^a^	32.60 ± 3.05 ^b^	23.60 ± 2.51 ^c^
Day 14	0.00 ± 0.00 ^d^	0.00 ± 0.00 ^d^	45.40 ± 4.34 ^a^	18.80 ± 2.59 ^b^	12.00 ± 3.54 ^c^

Data were expressed as mean ± S.D. Control group (received saline, orally); SMC50 (treated with s-Methylcysteine, 50 mg/kg, orally); C + ve (infected with 10^4^ *C. parvum* oocysts); C + SMC25 (infected and treated with s-Methylcysteine, 25 mg/kg, orally); c: C + SMC50 (infected and treated with s-Methylcysteine, 50 mg/kg, orally). Means within the same row (in each parameter) carrying different superscripts (a, b, c, d) are significantly different (*p* < 0.05).

**Table 2:** Serum liver biomarkers following s-Methylcysteine treatment in *C. parvum*-infected mice.

Serum Biochemical Parameters	Experimental Groups				
	Control	SMC50	C + ve	C + SMC25	C + SMC50
ALT (U/L)	42.72 ± 2.08 ^d^	39.28 ± 3.52 ^d^	109.22 ± 13.56 ^a^	71.18 ± 7.35 ^b^	53.35 ± 6.77 ^c^
AST (U/L)	58.48 ± 8.70 ^c^	54.34 ± 5.46 ^c^	151.68 ± 14.69 ^a^	83.96 ± 8.97 ^b^	58.52 ± 9.53 ^c^
ALP (U/L)	138.60 ± 13.43 ^cd^	130.60 ± 21.14 ^d^	240.20 ± 10.89 ^a^	179.00 ± 8.60 ^b^	80.154 ± 9.36 ^c^
Albumin (g/dL)	4.42 ± 0.32 ^a^	4.64 ± 0.23 ^a^	2.82 ± 0.19 ^d^	3.38 ± 0.33 ^c^	3.98 ± 0.19 ^b^
Globulin (g/dL)	4.88 ± 0.29 ^bc^	5.04 ± 0.21 ^b^	2.94 ± 0.18 ^d^	4.44 ± 0.58 ^c^	5.70 ± 0.36 ^a^

All data were expressed as mean ± S.D. Means within the same row (in each parameter) carrying different superscripts (a, b, c, d) are significantly different (*p* < 0.05). Control (received saline, orally); SMC50 (treated with s-Methylcysteine, 50 mg/kg, orally); C + ve (infected with 10^4^ *C. parvum* oocysts); C + SMC25 (infected and treated with s-Methylcysteine, 25 mg/kg, orally); C + SMC50 (infected and treated with s-Methylcysteine, 50 mg/kg, orally). ALT, alanine aminotransferase; AST, aspartate aminotransferase; ALP, alkaline phosphatase.

## Abbreviations

*Cryptosporidium parvum*	*C. parvum*
SMC	s-Methylcysteine
SMC25	s-Methylcysteine (25 mg/kg b.w.)
SMC50	s-Methylcysteine (50 mg/kg b.w.)
PI	post-infection
ALT	alanine aminotransferase
AST	aspartate aminotransferase
ALP	alkaline phosphatase
SAP	serum amyloid P
TNF-α	tumor necrosis factor alpha.
IL-6	Interleukin-6
IFN-γ	Interferon gamma
MDA	malondialdehyde
GSH	glutathione
SOD	superoxide dismutase
